# Social participation and mortality according to company size of the longest‐held job among older men in Japan: A 6‐year follow‐up study from the JAGES

**DOI:** 10.1002/1348-9585.12216

**Published:** 2021-03-31

**Authors:** Satoru Kanamori, Naoki Kondo, Tomoko Takamiya, Hiroyuki Kikuchi, Shigeru Inoue, Taishi Tsuji, Yuko Kai, Go Muto, Katsunori Kondo

**Affiliations:** ^1^ Teikyo University Graduate School of Public Health Tokyo Japan; ^2^ Department of Preventive Medicine and Public Health Tokyo Medical University Tokyo Japan; ^3^ Department of Social Epidemiology and Global Health Graduate School of Medicine and School of Public Health Kyoto University Kyoto Japan; ^4^ Faculty of Health and Sport Sciences University of Tsukuba Tokyo Japan; ^5^ Physical Fitness Research Institute Meiji Yasuda Life Foundation of Health and Welfare Tokyo Japan; ^6^ Department of Hygiene Kitasato University School of Medicine Kanagawa Japan; ^7^ Department of Social Preventive Medical Sciences Center for Preventive Medical Sciences Chiba University Chiba Japan; ^8^ Center for Well‐being and Society Nihon Fukushi University Aichi Japan; ^9^ Center for Gerontology and Social Science National Center for Geriatrics and Gerontology Aichi Japan

**Keywords:** community participation, health status disparities, leisure activities, social environment, work

## Abstract

**Objectives:**

The purpose of this study was to examine the relationship between social participation (type/pattern) and mortality according to company size of the longest‐held job among older men in Japan who have worked in the company.

**Methods:**

Longitudinal data from the Japan Gerontological Evaluation Study were used in this study. Functionally independent individuals aged 65 years and older in Japan were surveyed. Work and community organizations (local community, hobbies, and sports) were used as social participation. A Cox proportional hazards model was used to calculate mortality hazard ratios.

**Results:**

Analysis was carried out on 19 260 participants. A total of 2870 deaths occurred during the 6‐year follow‐up period. Those in companies with 49 or fewer employees had the highest prevalence of work participation and the lowest participation in any community organization. Regardless of company size, the mortality risk was significantly lower for participants in any social participation (eg, the hazard ratio for participation in a hobby organization among those with a company size of 49 employees or fewer was 0.74, 95% CI: 0.65‐0.85) compared to nonparticipants whose company size was 49 or fewer employees.

**Conclusions:**

In Japan, although older men who have worked for small companies may have fewer benefits, their social participation may reduce their mortality risks. To avoid increasing health inequalities, it is necessary to create an environment in which they are more likely to participate in social activities.

## 1 INTRODUCTION

Reducing health inequalities is a global challenge identified by the WHO General Assembly in 2009.^1^ In Japan, which is the world's longest‐lived country, health inequalities are widening because of the widening socioeconomic inequalities in recent years.^2^, ^3^ The reduction in health inequalities in Japan is listed as one of the basic directions to be achieved in the second term of the National Health Promotion Plan in the twenty‐first century [Health Japan 21 (the second term)].^4^

Company size can be linked to health inequalities attributable to the differences in health services and social protections across companies.^5^ In Japan, there are structural differences in the access stemming from the Industrial Safety and Health Act^6^; for large companies, the Act mandates to have industrial physicians, but it is not so for the smallest: workplaces with less than 50 employees. In addition, smaller companies have less health and safety activities,^6^ lower average salaries,^7^ poorer lifestyles,^8^ and various health checkup results.^8^ Long‐term exposure to such differences in company size may cause health inequalities in old age. In fact, the smaller the size of the company where older men have worked the longest (company size of the longest‐held job), the higher the risk of both death^9^ and lesser instrumental activities of daily living.^10^ However, none of the relationships were found for women in these two longitudinal studies. These results suggest that the influence of the company size of the longest‐held job is limited to older men.

On the other hand, social participation of older adults is the key to successful aging.^11^ Previous cohort studies suggest that social participation such as working^12^ and participation in community organizations like local communities, hobbies, and sports^13^ reduce mortality risk. However, since the relationship between social relationships, including social participation and health outcomes, varies depending on the socioeconomic status,^14^, ^15^ the relationship between social participation and mortality risk may also differ depending on the company size of the longest‐held job which is closely associated with socioeconomic status. In terms of health inequalities,^16^ it is necessary to clarify whether social participation lowers mortality risk even among older men who have worked for long periods in small companies that are most disadvantaged for mortality risk.

The purpose of this study was to examine the relationship between social participation (type/pattern) and mortality according to the company size of the longest‐held job among older men in Japan who have worked in the company.

## 2 MATERIALS AND METHODS

### 2.1 Study design and participants

A population‐based prospective cohort study was conducted in Japan. It was based on a sample from the Japan Gerontological Evaluation Study (JAGES).^17^, ^18^ The survey was sent to 95 827 individuals over the age of 65 years who were not certified for long‐term care at baseline. It was carried out in 13 municipalities from August 2010 to January 2012. Participants were randomly selected from each municipality. The certification for long‐term care is based on a national standard for long‐term care assessment,^19^ and the local government records who have been certified. Among the participants who responded to the baseline survey, those with invalid responses for ID number, age, and/or gender were excluded. Participants were followed for almost 6 years (a maximum of 6.6 years). Those who did not answer the question about the company size of the longest‐held job, who responded “I don't know” or “I have never had a job” to the question about company size, or who responded “I have never had a job” to the question about the type of longest‐held job were excluded. In addition, agriculture/forestry/fishery workers are mostly self‐employed^20^, ^21^; therefore, they were excluded from the analysis. Of the remaining subjects, only men were included in the analysis.

This study was conducted in accordance with the Helsinki Declaration. Ethical approval for the study was obtained from the Nihon Fukushi University Ethics Committee (application number: 10‐05), the National Center for Geriatrics and Gerontology (application number: No. 992‐2), and Chiba University Ethics Committee (application number: No. 2493), with participants’ consent implied by the return of the questionnaires.

### 2.2 Measures

#### 2.2.1 Mortality outcome

Death records from 2010 to 2016 (maximum: 6.6 years) were used from the municipal government database of public long‐term care insurance.

#### 2.2.2 Social participation

##### Type of social participation

Participation in working^12^ and community organization such as local community, hobbies, and sports,^13^ which is related to decreased mortality risk, was used as social participation. Participants were asked, “What is your current working status?”. Choices were “I have a paid job”, “I am retired from my job”, and “I have never had a job.” Among these choices, “I have a paid job” was treated as “participation” in working. For neighborhood association or residents’ association (local community), leisure activity group (hobbies), and sports group or club (sports), the frequency of participation was asked. Choices were “almost every day”, “two or three times a week”, “once a week”, “once or twice a month”, “a few times a year”, or “never.” Among these choices, “almost every day”, “two or three times a week”, “once a week”, “once or twice a month”, and “a few times a year” were treated as “participation.”

##### Pattern of social participation

Based on previous studies on social participation,^22^, ^23^, ^24^ the four types of social participation were categorized as work and community organizations (local community, hobbies, and sports). The four patterns of social participation were then classified into the following four categories: (a) those who are only working are “work‐only”; (b) those who participate only in community organization (one or more of local community, hobbies, and sports) are “community organization‐only”; (c) those who are working and participating in both and community organizations were designated as “both participation”; and (d) those who did not participate in both were designated as “both not participation.”

#### 2.2.3 Company size of the longest‐held job

Based on Japanese General Social Surveys,^25^ the indicator for the company size of the longest‐held job was developed. Participants were asked, “Of all your jobs to date, about how many people worked in the entire company or organization where you were employed the longest?” Choices were 1‐9 employees, 10‐49 employees, 50‐499 employees, 500‐9999 employees, 10 000 employees or more, “I don't know”, and “I have never had a job.” To obtain statistical power, 1‐9 employees and 10‐49 employees were categorized as “49 employees or less”; and 500‐9999 employees and 10 000 employees or more were categorized as “500 or more.” The reason for using this classification is that occupational health physicians (possibly part‐time) and health supervisors in workplaces of 50 or more employees and full‐time occupational health physicians in workplaces of 500 employees who are constantly in hazardous work are required to be employed as per the Industrial Safety and Health Act in Japan.^26^

#### 2.2.4 Covariates

Based on previous studies on the relationship between social participation and mortality,^12^, ^13^ the following were used as covariates: age (65‐69 years, 70‐74 years, 75‐79 years, 80 years, and 85 years or more), annual equivalized income (less than 2 million yen per year  =  “low”; 2‐3.99 million yen per year  =  “middle”; or 4 million yen or more per year  =  “high”), educational attainment (less than 6 years, less than 9 years, 10‐12 years, or more than 12 years), type of longest‐held job (professional/technical or administrative, clerical or sales/service, skilled/labor, or others^10^), municipalities, household composition (living alone or with others), body mass index (BMI: less than 18.5, 18.5 to 24.9, or 25.0 or more), and self‐reported medical conditions (cancer, heart disease, stroke, hypertension, diabetes mellitus, hyperlipidemia, joint disease/neuralgia, respiratory disease, and psychiatric disease: yes or no for each medical condition).

### 2.3 Statistical analyses

Analysis of variance and chi‐square test were used to test the relationship between the company size of the longest‐held job and characteristics at baseline. The Cox proportional hazards model was used to calculate mortality hazard ratios (HRs). We created an independent variable that combined social participation with company size to conduct the analysis. At this time, the reference category was nonparticipants with a company size of 49 employees or fewer. Respondents who were lost to follow‐up because they relocated were censored. Model 1 was adjusted for age, annual equivalized income, educational attainment, type of longest‐held job, municipalities, household composition, BMI, and self‐reported medical conditions (cancer, heart disease, stroke, hypertension, diabetes mellitus, hyperlipidemia, joint disease/neuralgia, respiratory disease, and psychiatric disease). Model 2 was adjusted for the covariates in Model 1 plus the company size of the longest‐held job. Furthermore, a sensitivity analysis, the analysis excluding those who died within 2 years, was performed to reduce the possibility of reverse causation. They were more likely to be functionally impaired to the extent that they could not participate in social activities at the time of the survey, and excluding them weakened the possibility of reverse causation.

Missing data were replaced with a dummy variable. Statistical significance was set at *P* < .05. All statistical analyses were conducted using IBM SPSS version 21.0.

## 3 RESULTS

**FIGURE 1:**
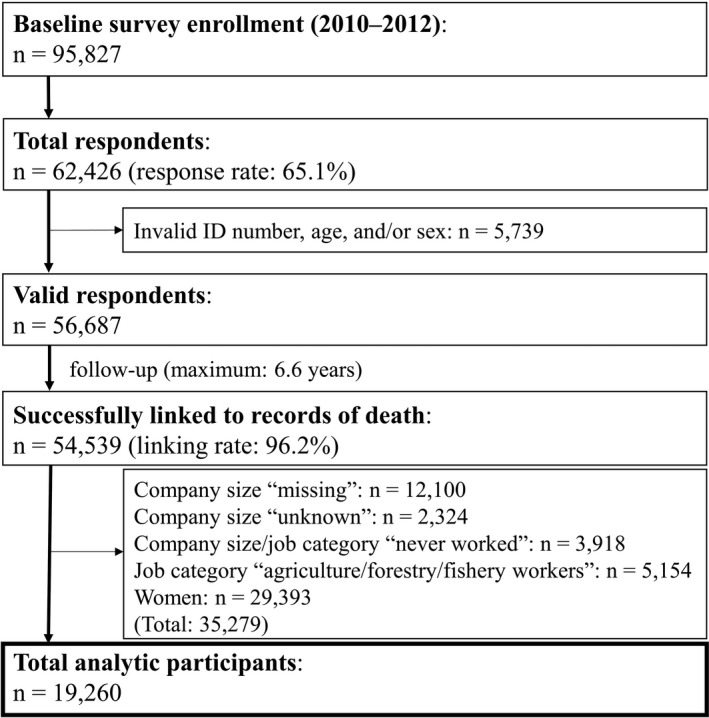
Flowchart of participants

Figure 1 shows a flowchart of participants. Of the 95 827 individuals included in the baseline survey, responses were received from 62 426 individuals. Of these subjects, those who met the exclusion criteria were removed, and the remaining 19 260 subjects were analyzed. The number of people according to the company size of the longest‐held job was 7487 (38.9%) for 49 or fewer employees, 5285 (27.4%) for 50‐499 employees, and 6488 (33.7%) for 500 or more employees. The average follow‐up period was 5.5 ± 1.3 years, 5.5 ± 1.3 years, and 5.5 ± 1.2 years, respectively. The mortality rate per 1000 person‐years was 28.0, 25.4, and 22.8, respectively.

Table 1 shows the characteristics of participants according to the company size of the longest‐held job. Significant differences were found between company sizes in all variables, except age and a part of self‐reported medical conditions. Similarly, significant differences between company sizes were observed in any type and pattern of social participation. The highest prevalence of participation in work and the lowest prevalence of participation in each community organization were found in the group of 49 or fewer. With regard to the pattern of social participation, the largest difference between company sizes was in the prevalence of participation in “community organizations‐only.”

**TABLE 1:** Characteristics of participants according to the company size of the longest‐held job

	Company size of the longest‐held job (number of employees)								*P*
	Total		−49		50‐499		500+		
	(N = 19 260)		(N = 7487)		(N = 5285)		(N = 6488)		
	N	%	N	%	N	%	N	%	
Age									
Mean ± SD	73.3 ± 5.7		73.4 ± 5.7		73.2 ± 5.7		73.2 ± 5.8		.07
Annual equivalized income									
Low	7633	39.6	3500	46.7	2184	41.3	1949	30.0	<.001
Middle	7570	39.3	2304	30.8	2003	37.9	3263	50.3	
High	2071	10.7	759	10.1	522	9.9	790	12.2	
Educational attainment									
Less than 6 y	221	1.1	128	1.7	56	1.1	37	0.6	<.001
6‐9 y	7457	38.7	3691	49.3	2074	39.2	1692	26.1	
10‐12 y	6497	33.7	2120	28.3	1862	35.2	2515	38.8	
13 y or more	4817	25.0	1433	19.1	1224	23.2	2160	33.3	
Type of longest‐held job									
Professional/technical or administrative	7321	38.0	2504	33.4	1897	35.9	2920	45.0	<.001
Clerical or sales/service	4903	25.4	1834	24.5	1459	27.6	1610	24.8	
Skilled/labor	3969	20.6	1562	20.9	1116	21.1	1291	19.9	
Other	1899	9.9	1067	14.3	487	9.2	345	5.3	
Household composition									
Living alone	1435	7.4	619	8.3	436	8.2	380	5.9	<.001
With others	17 590	91.3	6762	90.3	4790	90.6	6038	93.1	
Body mass index									
Less than 18.5	1065	5.5	425	5.7	303	5.7	337	5.2	<.001
18.5‐24.9	13 596	70.6	5103	68.2	3797	71.8	4696	72.4	
25.0 or more	4253	22.1	1768	23.6	1098	20.8	1387	21.4	
Self‐reported medical conditions									
Cancer	1193	6.2	435	5.8	338	6.4	420	6.5	.261
Heart disease	2818	14.6	1109	14.8	784	14.8	925	14.3	.488
Stroke	376	2.0	161	2.2	87	1.6	128	2.0	.131
Hypertension	7274	37.7	2840	37.9	2004	37.9	2430	37.5	.525
Diabetes mellitus	2956	15.3	1145	15.3	848	16.0	963	14.8	.309
Hyperlipidemia	1493	7.7	496	6.6	377	7.1	620	9.6	<.001
Joint disease/Neuralgia	1384	7.2	605	8.1	368	7.0	411	6.3	<.001
Respiratory disease	890	4.6	359	4.8	284	5.4	247	3.8	<.001
Psychiatric disease	143	0.7	56	0.7	47	0.9	40	0.6	.270
Type of social participation									
Work	5154	26.8	2702	36.1	1223	23.1	1229	18.9	<.001
Local community	8218	42.7	2870	38.3	2372	44.9	2976	45.9	<.001
Hobbies	7688	39.9	2571	34.3	2020	38.2	3097	47.7	<.001
Sports	4866	25.3	1521	20.3	1326	25.1	2019	31.1	<.001
Pattern of social participation^a^									
Both not participation	3471	18.0	1342	17.9	1001	18.9	1128	17.4	<.001
Work only	1358	7.1	787	10.5	321	6.1	250	3.9	
Community organizations only	8265	42.9	2340	31.3	2410	45.6	3515	54.2	
Both participation	3301	17.1	1623	21.7	783	14.8	895	13.8	

Note

The analysis of variance and chi‐square test were used to test the relationship between the company size of the longest‐held job and characteristics.

Missing values for social participation have been omitted.

^a^ Pattern of social participation: “work‐only” means those who are only working; “community organization‐only” means those who participate only in community organization (one or more of the local community, hobbies, and sports); “both participation” means those who participated in both and community organizations; and “both not participation” means those who did not participate in both.

**FIGURE 2:**
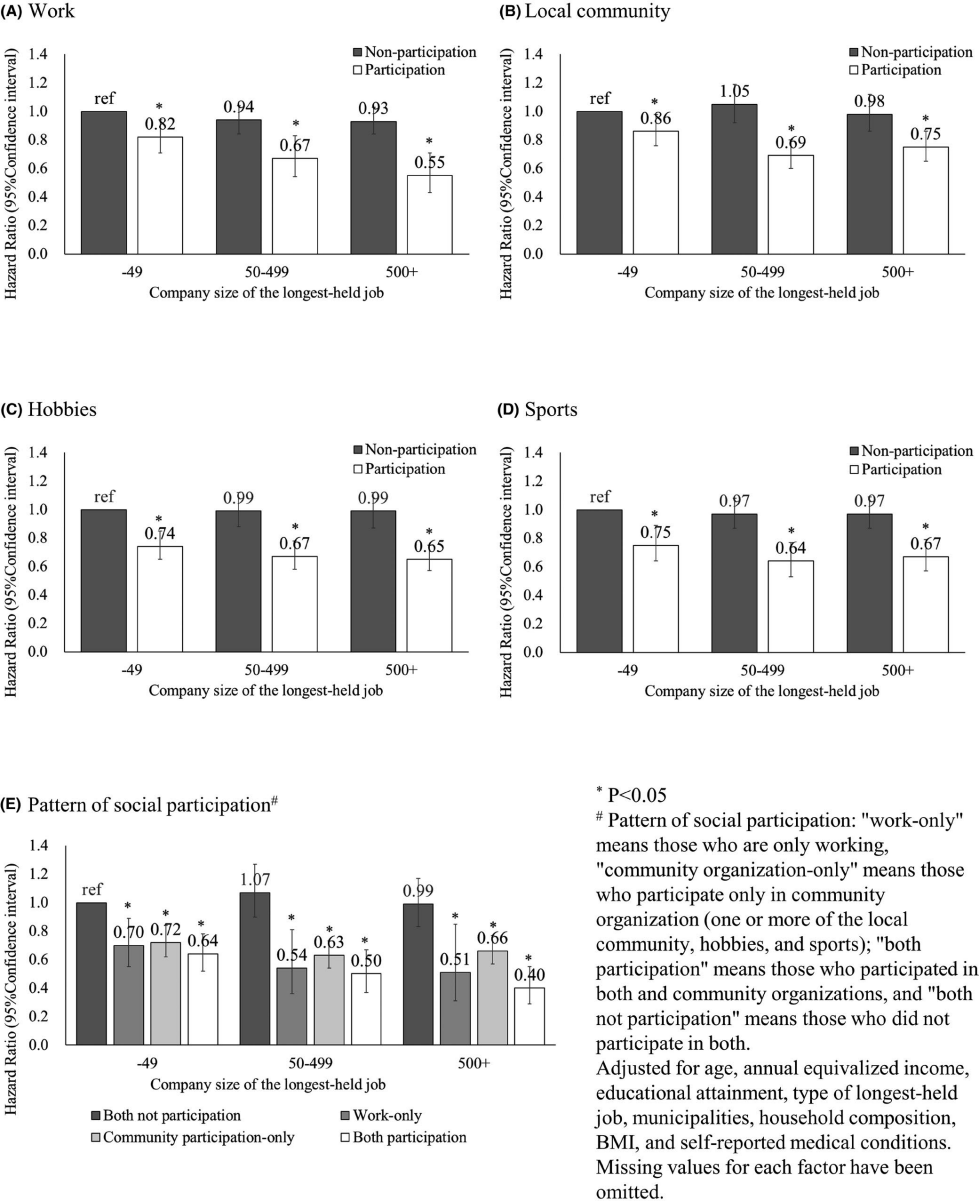
Mortality hazard ratios for social participation according to the company size of the longest‐held job

Figure 2 and Appendix S1 show mortality HRs for social participation according to the company size of the longest‐held job. Participants in the work had a significantly lower mortality risk regardless of company size compared to nonparticipants with a company size of 49 employees or fewer. Similar relationships were also found in local communities, hobbies, and sports. However, among those with a company size of 49 employees or fewer, the significant difference in mortality HRs in only participants in work (HR = 0.87; 95% confidence interval = 0.74‐1.02) and local community (0.90; 0.77‐1.05) disappeared in the sensitivity analysis. Regarding the pattern of social participation, “work‐only,” “community organization‐only,” and “both participation” in any company size had significantly lower mortality risk than “both not participation” in a company size of 49 employees or fewer. In the sensitivity analysis, the significant difference in mortality HRs disappeared only for “work‐only” whose company size was 49 employees or fewer (0.78; 0.60‐1.03).

## 4 DISCUSSION

This study examined the relationship between social participation (type/pattern) and mortality according to the company size of the longest‐held job. Participants performing all types and patterns of social activities had a significantly lower risk of mortality regardless of company size compared to nonparticipants working in a company with less than 49 employees. There was no statistical difference in mortality risk between company sizes in the nonparticipation group for any type or pattern of social participation. However, the reduction in mortality risk in the participation group was smallest in those with a company size of 49 employees or fewer.

Previous studies have shown that participation in work and community participation are associated with a lower mortality risk among older adults,^12^, ^13^ and the results of this study are consistent with those of the previous studies. The present study also found that any social participation was associated with a lower mortality risk, even among socially disadvantaged groups of less than 49 employees. However, in a sensitivity analysis that excluded deaths within 2 years, significant differences disappeared for participation in work (0.87; 0.74‐1.02), local community (0.90; 0.77‐1.05), and work only (0.78; 0.60‐1.03) in the group of 49 or fewer employees. The 95% confidence interval for these HRs may have slightly straddled 1 because of the reduction in statistical power by increasing the categories according to company size. Alternatively, those who worked at small companies could have participated in these organizations despite their ill health. Future studies should clarify these relationships by extending the sample size and follow‐up period.

The reduction in mortality risk because of any social participation was smallest among those with a company size of 49 employees or fewer. This suggests that the health benefits of social participation were less for those who worked in small companies than for those who worked in large companies. A previous cohort study among older Japanese adults showed that those with longer education have a lower risk of functional disability because of social participation than those with shorter education.^15^ The trend in the results of the present study is consistent with the findings of this previous study. Since it has been shown that those with higher levels of education can accumulate health capital more effectively than those with lower levels of education,^27^ the low mortality risk because of social participation may have been smaller in a group of small companies with a greater number of those with shorter education histories.

Among our study participants, those who worked in the workplace with 49 or fewer workers were the most likely to continue working but the least likely to participate in community organizations (local community, hobbies, and sports). In the present study, educational attainment and income were associated with company size, and these factors may contribute to the association between company size and participation in community organizations. This is in concordance with other studies in Japan that observed different age groups. For example, previous research on Japanese workers aged 55‐59 years shows that those who work for larger companies are less willing to work at older ages.^28^ Results of this previous study support the differences in the proportion of work participation across company sizes in this study. A new finding in this study is that the company size of the longest‐held job is associated with working status in older age. It has also been shown that those who do not own a home, have a mortgage, or have small savings accounts are more willing to work longer.^28^ Average income is lower for smaller companies,^7^ which may reflect the fact that employees working in smaller groups of companies are forced to work even after retirement. On the other hand, previous study with middle‐aged Japanese adults has shown that education attainment and lifestyle orientation are positively associated with social participation in sports and leisure activities.^22^ Previous study among older Japanese adults has also shown that education attainment and income are positively associated with participation in sports groups.^29^

There are several implications for occupational and community health practitioners and policymakers. At the individual level, one of them is to encourage some forms of social participation (either through work or community organizations) in old age. At the policy level, a perspective of proportionate universalism, which strengthens measures in proportion to the weakness of the social position, is important.^30^ Older men who have worked in smaller companies have a higher mortality risk compared to older men who have worked in larger companies and are less likely to participate in community organizations that may reduce their mortality risk. Furthermore, the health benefits of social participation for men who worked in small companies may be smaller than for men who worked in large companies, and it is feared that the current approach contributes to increasing health inequalities. Therefore, it is particularly important to create an environment that encourages their participation. In addition, since social participation in old age is associated with community involvement from middle age,^31^ a preretirement approach is also key. In Japan, the guidelines for promoting cooperation between organizations in charge of community health and those responsible for employees’ health were revised in 2019.^32^ One of the initiatives is to promote engagement with under‐supported segments of the population, such as small companies. The implications of this study should be reflected in such measures.

This study has several limitations. The first is that it does not necessarily reflect the overall picture of men who have worked in the company among the surveyed population. This is because the response rate of the questionnaire was 65.1%, and it was not possible to accurately disaggregate those who had worked in the company. It is possible that those whose longest‐held jobs were self‐employed or government employees were included in the analysis, although we excluded agriculture/forestry/fishery workers, who are often self‐employed, from the analysis. Second, survival bias may have affected the results of this study. The risk of mortality was higher among older men who have worked for small companies, or among those who did not participate in work or community organizations. They are more likely than others to die prior to the study and may have been selectively omitted from the study population. For this reason, the mortality risk from social participation among older men who have worked for small companies may be underestimated. Third, there may be a nondiscriminatory misclassification in the measure of the company size of the longest‐held job. The questions on company size and job type may have been difficult to answer accurately because they asked about previous long‐term employment. Therefore, the relationships observed in this study may have been underestimated. Fourth, we were unable to use information on causes of death and could not identify which causes of death social participation may specifically reduce the risk of death. Hence, we are unable to infer a mechanism for reducing mortality and differences between company sizes in the relationship between social participation and mortality risk.

In conclusion, regardless of the company size of the longest‐held job (even if the company with fewer than 49 employees), older men in Japan who have worked in a company may be able to reduce their mortality risk through social participation, especially in hobbies and sports. However, the smaller the company size of the longest‐held job, the smaller the benefit is likely to be. To avoid increasing health inequalities in old age according to the company size of the longest‐held job, it is necessary to create an environment in which people who have worked in smaller companies are more likely to participate in social activities.

## CONFLICT OF INTEREST

Authors declare no conflict of interests for this article.

## DISCLOSURES

*Approval of the research protocol*: The Research Ethics Committee of the Nihon Fukushi University Ethics Committee (application number: 10‐05) reviewed and approved the aims and procedures of this study. *Informed consent*: Informed consent was obtained from all individual participants included in the study. *Registry and the registration no. of the study/trial*: N/A. *Animal studies*: N/A.

## AUTHOR CONTRIBUTIONS

SK conducted the analysis and wrote manuscript in collaboration with NK, T Takamiya, HK, and SI; and T Tsuji wrote the first draft of the manuscript. YK, GM, and KK provided the feedback and suggestions. All authors read the manuscript and approved to submission.

## ETHICAL APPROVAL

Ethical approval for the study was obtained from the Nihon Fukushi University Ethics Committee (application number: 10‐05). This study was performed in accordance with the principles of the Declaration of Helsinki. Informed consent was obtained from all participants.

## Supporting information

Appendix S1
