# Supplementary material for: Social participation and mortality according to company size of the longest‐held job among older men in Japan: A 6‐year follow‐up study from the JAGES
Source: J Occup Health. 2021 Mar 31;63(1):e12216. doi: 10.1002/1348-9585.12216 (PMC8012407; doi:10.1002/1348-9585.12216)
Supplement: Supplementary file 1 — Appendix S1 [file JOH2-63-e12216-s001.docx]

**APPENDICES**

Appendix S1. Mortality hazard ratios for social participation according to the company size of the longest-held job

|  | Company size | Participation | N | Deaths | Person -years | Crude | | Model 1 | |
| --- | --- | --- | --- | --- | --- | --- | --- | --- | --- |
|  |  |  |  |  |  | HR | 95%CI | HR | 95%CI |
| **Type of social participation** | | | | | | | | | |
| Work | -49 | Non-participation | 4,297 | 824 | 23,106 | ref |  | ref |  |
|  |  | Participation | 2,702 | 282 | 15,209 | 0.52 | 0.45-0.59 | 0.82 | 0.71-0.94 |
|  | 50-499 | Non-participation | 3,795 | 636 | 20,639 | 0.86 | 0.78-0.96 | 0.94 | 0.84-1.04 |
|  |  | Participation | 1,223 | 93 | 6,894 | 0.38 | 0.30-0.47 | 0.67 | 0.54-0.83 |
|  | 500+ | Non-participation | 5,053 | 761 | 27,461 | 0.78 | 0.70-0.86 | 0.93 | 0.84-1.03 |
|  |  | Participation | 1,229 | 68 | 6,973 | 0.27 | 0.21-0.35 | 0.55 | 0.43-0.71 |
| Local community | -49 | Non-participation | 3,331 | 589 | 17,827 | ref |  | ref |  |
|  |  | Participation | 2,870 | 390 | 16,121 | 0.73 |  | 0.86 | 0.76-0.98 |
|  | 50-499 | Non-participation | 2,170 | 374 | 11,592 | 0.98 | 0.86-1.11 | 1.05 | 0.92-1.19 |
|  |  | Participation | 2,372 | 269 | 13,406 | 0.60 | 0.53-0.69 | 0.69 | 0.60-0.80 |
|  | 500+ | Non-participation | 2,749 | 413 | 14,713 | 0.85 | 0.75-0.97 | 0.98 | 0.86-1.12 |
|  |  | Participation | 2,976 | 311 | 16,838 | 0.55 | 0.48-0.63 | 0.75 | 0.65-0.86 |
| Hobbies | -49 | Non-participation | 3,689 | 653 | 19,894 | ref |  | ref |  |
|  |  | Participation | 2,571 | 325 | 14,349 | 0.69 | 0.60-0.78 | 0.74 | 0.65-0.85 |
|  | 50-499 | Non-participation | 2,545 | 436 | 13,730 | 0.97 | 0.86-1.09 | 0.99 | 0.88-1.12 |
|  |  | Participation | 2,020 | 226 | 11,357 | 0.60 | 0.52-0.70 | 0.67 | 0.58-0.78 |
|  | 500+ | Non-participation | 2,707 | 435 | 14,651 | 0.90 | 0.80-1.02 | 0.99 | 0.87-1.12 |
|  |  | Participation | 3,097 | 298 | 17,325 | 0.52 | 0.45-0.60 | 0.65 | 0.57-0.75 |
| Sports | -49 | Non-participation | 4,629 | 790 | 25,171 | ref |  | ref |  |
|  |  | Participation | 1,521 | 179 | 8,518 | 0.67 | 0.57-0.79 | 0.75 | 0.64-0.89 |
|  | 50-499 | Non-participation | 3,142 | 509 | 17,080 | 0.95 | 0.85-1.06 | 0.97 | 0.87-1.08 |
|  |  | Participation | 1,326 | 130 | 7,515 | 0.55 | 0.46-0.66 | 0.64 | 0.53-0.77 |
|  | 500+ | Non-participation | 3,681 | 542 | 20,127 | 0.86 | 0.77-0.96 | 0.97 | 0.87-1.09 |
|  |  | Participation | 2,019 | 179 | 11,281 | 0.50 | 0.43-0.59 | 0.67 | 0.57-0.79 |
|  |  |  |  |  |  |  |  |  |  |
| **Pattern of social participation^#^** | | | | | | | | | |
|  | -49 | Both not participation | 1,342 | 325 | 6,951 | ref |  | ref |  |
|  |  | Work-only | 787 | 87 | 4,377 | 0.42 | 0.33-0.53 | 0.70 | 0.55-0.89 |
|  |  | Community organizations-only | 2,340 | 368 | 12,886 | 0.60 | 0.52-0.70 | 0.72 | 0.62-0.84 |
|  |  | Both participation | 1,623 | 156 | 9,232 | 0.36 | 0.29-0.43 | 0.64 | 0.52-0.78 |
|  | 50-499 | Both not participation | 1,001 | 231 | 5,182 | 0.95 | 0.81-1.13 | 1.07 | 0.90-1.27 |
|  |  | Work-only | 321 | 25 | 1,782 | 0.30 | 0.20-0.45 | 0.54 | 0.36-0.81 |
|  |  | Community organizations-only | 2,410 | 324 | 13,435 | 0.51 | 0.44-0.59 | 0.63 | 0.54-0.73 |
|  |  | Both participation | 783 | 53 | 4,479 | 0.25 | 0.19-0.33 | 0.50 | 0.37-0.67 |
|  | 500+ | Both not participation | 1,128 | 239 | 5,877 | 0.87 | 0.74-1.03 | 0.99 | 0.83-1.17 |
|  |  | Work-only | 250 | 16 | 1,386 | 0.24 | 0.15-0.40 | 0.51 | 0.31-0.85 |
|  |  | Community organizations-only | 3,515 | 429 | 19,496 | 0.47 | 0.40-0.54 | 0.66 | 0.57-0.77 |
|  |  | Both participation | 895 | 44 | 5,128 | 0.18 | 0.13-0.25 | 0.40 | 0.29-0.55 |

^#^ Pattern of social participation: “work-only” means those who are only working; “community organization-only” means those who participate only in community organization (one or more of the local community, hobbies, and sports); “both participation” means those who participated in both and community organizations; and “both not participation” means those who did not participate in both.

Model 1 was adjusted for age, annual equivalized income, educational attainment, type of longest-held job, municipalities, household composition, BMI, and self-reported medical conditions.

Missing values for social participation have been omitted.
